# Temporal characteristics of astrocytic activation in the TNC in a mice model of pain induced by recurrent dural infusion of inflammatory soup

**DOI:** 10.1186/s10194-021-01382-9

**Published:** 2022-01-15

**Authors:** Leyi Zhang, Chenglong Lu, Li Kang, Yingji Li, Wenjing Tang, Dengfa Zhao, Shengyuan Yu, Ruozhuo Liu

**Affiliations:** 1 Medical School of Chinese PLA, Beijing, 100853 People’s Republic of China; 2 Department of Neurology, the First Medical Center, Chinese PLA General Hospital, Fuxing Road 28, Haidian District, Beijing, 100853 People’s Republic of China

**Keywords:** Chronic migraine, Astrocytic activation, Temporal characteristics, Cytokines, Trigeminal nucleus caudalis

## Abstract

**Background:**

Astrocytic activation might play a significant role in the central sensitization of chronic migraine (CM). However, the temporal characteristics of the astrocytic activation in the trigeminal nucleus caudalis (TNC) and the molecular mechanism under the process remain not fully understood. Therefore, this study aims to investigate the duration and levels change of astrocytic activation and to explore the correlation between astrocytic activation and the levels change of cytokines release.

**Methods:**

We used a mice model induced by recurrent dural infusion of inflammatory soup (IS). The variation with time of IS-induced mechanical thresholds in the periorbital and hind paw plantar regions were evaluated using the von Frey filaments test. We detected the expression profile of glial fibrillary acidic protein (GFAP) in the TNC through immunofluorescence staining and western blot assay. We also investigated the variation with time of the transcriptional levels of GFAP and ionized calcium binding adapter molecule 1 (Iba1) through RNAscope in situ hybridization analysis. Then, we detected the variation with time of cytokines levels in the TNC tissue extraction and serum, including c-c motif chemokine ligand 2 (CCL2), c-c motif chemokine ligand 5 (CCL5), c-c motif chemokine ligand 7 (CCL7), c-c motif chemokine ligand 12 (CCL12), c-x-c motif chemokine ligand 1 (CXCL1), c-x-c motif chemokine ligand 13 (CXCL13), interferon gamma (IFN-γ), tumor necrosis factor alpha (TNF-α), macrophage colony-stimulating factor (M-CSF), interleukin 1beta (IL-1β), interleukin 6 (IL-6), interleukin 10 (IL-10), interleukin 17A (IL-17A).

**Results:**

Recurrent IS infusion resulted in cutaneous allodynia in both the periorbital region and hind paw plantar, ranging from 5 d (after the second IS infusion) to 47 d (28 d after the last infusion) and 5 d to 26 d (7 d after the last infusion), respectively. The protein levels of GFAP and messenger ribonucleic acid (mRNA) levels of GFAP and Iba1 significantly increased and sustained from 20 d to 47 d (1 d to 28 d after the last infusion), which was associated with the temporal characteristics of astrocytic activation in the TNC. The CCL7 levels in the TNC decreased from 20 d to 47 d. But the CCL7 levels in serum only decreased on 20 d (1 d after the last infusion). The CCL12 levels in the TNC decreased on 22 d (3 d after the last infusion) and 33 d (14 d after the last infusion). In serum, the CCL12 levels only decreased on 22 d. The IL-10 levels in the TNC increased on 20 d.

**Conclusions:**

Our results indicate that the astrocytic activation generated and sustained in the IS-induced mice model from 1 d to 28 d after the last infusion and may contribute to the pathology through modulating CCL7, CCL12, and IL-10 release.

## Background

Migraine is a severe neurological disorder with high morbidity (13.8%–15.0%) and disability (2nd most) [1]. Chronic migraine (CM), transformed from episodic migraine, is much severer and the mechanisms underlying the progression are not fully understood. It has been suggested that the high frequency of migraine attacks leads to CM, which involves central sensitization of the trigeminovascular system and neurogenic neuroinflammation [2–4].

Central sensitization refers to increased neuronal excitability and synaptic plasticity of central neurons in the trigeminal nociceptive pathway, especially the trigeminal nucleus caudalis (TNC), which are brainstem trigeminal neurons, or the second-order of the trigeminovascular system [5, 6]. Central sensitization is manifested as cutaneous allodynia, which means an enlarged area of hyperalgesia [7]. It has been suggested that activation of the astrocytes surrounding the TNC neurons are directly or indirectly involved in central sensitization [8]. Astrocytic activation upregulates glial fibrillary acidic protein (GFAP) and releases several cytokines, which increases neuronal excitability and contributes to the sensitization process. GFAP is thought to be an important biomarker of astrocytic activation. Altered levels of pro-inflammation cytokines in plasma and serum, such as interleukin 1 beta (IL-1β), interleukin 6 (IL-6), and tumor necrosis factor alpha (TNF-α), has been demonstrated the correlation to migraine in both patients and animals [9–12]. But it remains unclear whether the levels change of cytokines in the TNC tissue and serum are correlated to the central sensitization and astrocytic activation in the TNC region in CM. The temporal characteristics of astrocytic activation in the TNC have not been fully understood.

In this study, we investigated the variation with time of cutaneous allodynia behavior and the temporal characteristics of astrocytic activation in the TNC in a mice model of pain induced by recurrent dural infusion of inflammatory soup (IS), which could well simulate some elements of the CM pathology [13]. In this model, we explored the protein and messenger ribonucleic acid (mRNA) levels change of GFAP in the TNC. We also explored the mRNA levels change of ionized calcium binding adapter molecule 1 (Iba1), a biomarker of microglial activation. Then, we examined the levels change of 13 cytokines, including c-c motif chemokine ligand 2 (CCL2), c-c motif chemokine ligand 5 (CCL5), c-c motif chemokine ligand 7 (CCL7), c-c motif chemokine ligand 12 (CCL12), c-x-c motif chemokine ligand 1 (CXCL1), c-x-c motif chemokine ligand 13 (CXCL13), interferon gamma (IFN-γ), TNF-α, macrophage colony-stimulating factor (M-CSF), IL-1β, IL-6, interleukin 10 (IL-10), interleukin 17A (IL-17A), which could correlate with CM, both in the TNC tissue extraction and serum.

## Materials and methods

### Animals

Male adult C57BL/6 J mice (21–34 g) used in the experiments were obtained from SPF Biotechnology Co., Ltd. (Beijing, China). The mice were kept in individual cages with a room temperature of 23 ± 2 °C, the humidity of 50 ± 10%, a 12 h light-dark cycle, food and water ad libitum. All procedures were approved by the Institutional Animal Care and Use Committee at the Chinese PLA General Hospital and followed Regulations for The Administration of Affairs Concerning Experimental Animals. After a week of adaption, the animals were randomly assigned to experimental groups.

### Surgical procedure

A cannula was implanted to the skull of each mouse for IS or normal saline (NS) infusion with reference to other research [13, 14]. Briefly, a mouse was anesthetized intraperitoneally (i.p.) with 1% pentobarbital sodium (7.5 ml/Kg) and placed onto a stereotaxic frame. The animal temperature was maintained during surgery using a constant temperature heating blanket. After hair removal and disinfection of the surgical area, a 5- to 8-mm-long midline incision was made to expose bregma. A burr hole, 1.5 mm caudal to bregma, 1.5 mm lateral to the midline, adjacent to the superior sagittal sinus, was slowly drilled to expose the dura mater, taking care not to pierce the dura. A small screw (M 1.0 mm, L 2.0 mm) was invertedly affixed to the site, approximately 3 mm contralateral to the midline, by using biomedical glue. A guide cannula (O.D. 0.41 mm, I.D. 0.25 mm, length 0.3 mm, RWD, China) filled with a dummy cannula (O.D. 0.20 mm, length 0.4 mm) was implanted to the drilled hole and affixed to the skull by applying dental cement to cover the foundation of the cannula and the screw. A microsyringe (10 μL, Hamilton), an injection cannula (O.D. 0.21 mm, I.D. 0.11 mm), and a length of polyethylene tube were assembled into a drug delivery system. It was used to deliver IS or NS to the dura mater. After surgery, the mouse was returned to its cage when it was fully awake and recovered for at least 7 days before the next procedure. Analgesics were not used during the recovering period due to the concern of any interference to the pain pattern of the animal model.

### Recurrent IS or NS infusion

**Fig. 1:**
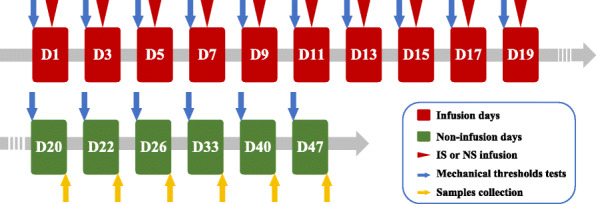
Experimental flow chart for studying the temporal characteristics of astrocytic activation in the TNC and the release of the cytokines in the TNC tissue extraction and serum. IS: inflammatory soup. NS: normal saline. D: day

56 mice were separated into 7 groups (*n* = 8) at random: (1) IS-20 group (IS infusion, sacrificed on 20 d), (2) IS-22 group (IS infusion, sacrificed on 22 d), (3) IS-26 group (IS infusion, sacrificed on 26 d), (4) IS-33 group (IS infusion, sacrificed on 33 d), (5) IS-40 group (IS infusion, sacrificed on 40 d), (6) IS-47 group (IS infusion, sacrificed on 47 d), and (7) control (CON) group (NS infusion, sacrificed on 47 d). IS contains 1 mM histamine, 1 mM serotonin, 1 mM bradykinin, and 0.1 mM prostaglandin E2 in NS. All procedures took place between 8:00 and 18:00. On infusion days, after the dummy cannula was removed from the guide cannula, mice in IS groups were received 5 μL IS infusion by the drug delivery system through the guide cannula. Mice in the CON group received the same dose of NS instead. The infusion was operated for a total of 10 times on alternate days (days 1, 3, 5, 7, 9, 11, 13, 15, 17, 19). It needs to be noted that the mice in the CON group only followed the same timeline of the IS-47 group and were sacrificed on 47 d (Fig. 1)

### Cutaneous allodynia behavior

Cutaneous allodynia behavior of mice was evaluated with mechanical thresholds in the periorbital region and the hind paw plantar, as described previously [15, 16]. The animals were habituated in a plexiglass box with a mesh grid bottom for 15–20 min before measurement. Mechanical thresholds were measured by applying von Frey filaments (vFF) perpendicularly to the periorbital region and the hind paw plantar, with the filaments bending for approximately three seconds or until a positive response is evoked. The vFF is calibrated by the manufacturer. The force range of the vFF applied to the periorbital region and the hind paw plantar is 0.008–0.4 g (0.008, 0.02, 0.04, 0.07, 0.16, 0.4 g) and 0.07–4 g (0.07, 0.16, 0.4, 0.6, 1.0, 1.4, 2.0, 4.0 g), respectively. The experimenter applied the filaments with sequential increasing order to determine the mechanical thresholds. After a positive response is evoked, the next filament of less force is applied. And if it is a negative response, the next filament of higher force will be applied. The positive response is considered as the animal quickly retracts its head or rubs its face in the measurement of mechanical thresholds in the periorbital region. In the measurement of mechanical thresholds in the hind paw plantar, the positive response is considered as the animal licks, retracts, or shakes its hind paw. The mechanical thresholds are defined as the force which would firstly evoke a positive response three times in five or more trials. Only the mice in the IS-47 group (*n* = 8) and CON group (*n* = 8) were performed the measurement. The mechanical thresholds were measured before each IS/NS infusion (days 1, 3, 5, 7, 9, 11, 13, 15, 17, 19). On days 20, 22, 26, 33, 40, and 47, we also take mechanical thresholds tests at the same time of day as before (8:00–18:00). The mechanical thresholds measured on day 1 were regarded as a baseline. The experimenter was blinded to the grouping during the tests.

### Sample collection

The serum and the TNC tissue of each animal were collected. On the sample collection day (8:00–18:00), the animals in each group were sacrificed under deep anesthesia with 1% pentobarbital sodium. Four mice of each group were taken blood samples from the vein near the eye into Eppendorf (EP) tubes. After 20-min undisturbed incubation and 10-min centrifugation (3000 rpm, 4 °C), the serum was aliquoted and stored at − 80 °C for subsequent analysis. The brains were separated from the skull quickly on ice after transcardial phosphate-buffered solution (PBS) perfusion. Four brains of each group were embedded with optimum cutting temperature compound (OCT, Tissue-Tek), snap-frozen in liquid nitrogen, and then stored at − 80 °C for the next sectioning procedure. The TNC tissue of the other four brains in each group was immediately isolated, snap-frozen in liquid nitrogen, and stored at − 80 °C for the following procedures. The integrity of the dura mater was visually inspected.

### Immunofluorescent staining

The brains (*n* = 4/group) embedded with OCT were manufactured into 10-μm-thick frozen sections of TNC tissue in a cryostat microtome (Leica 1950 M). After fixation in ice-cold acetone, the sections were blocked by blocking buffer (10% normal goat serum, 0.5% Triton X-100, dissolved in 0.1 M PBS) for 1 h at room temperature (RT). Then, the sections were incubated with primary antibody (rabbit anti-GFAP antibody, 1:100, ab207165, Abcam) diluted in primary antibody dilution buffer (P0262, Beyotime) overnight at 4 °C. After cleaning with 0.1 M PBS three times, the sections were incubated with secondary antibody (Alexa Fluor 488-conjugated goat anti-rabbit IgG, 1:1000, ab150077, Abcam) diluted in secondary antibody dilution buffer (P0265, Beyotime) for 1 h at RT. Then, the sections were rinsed in 0.1 M PBS three times and mounted with an antifade mounting medium with 4′,6-diamidino-2-phenylindole (DAPI, P0131, Beyotime). Magnified Images (× 20 objective) were captured under a fluorescence microscope (BX43, Olympus) using the cellSens standard software (version 1.18, Olympus). In negative-control sections, PBS was used instead of primary antibody, and there were no positive signals. The immunofluorescence area fraction was measured using ImageJ software (version 1.52p, National Institutes of Health).

### Western blot assay

We examined the protein levels of GFAP through a western blot assay. The TNC tissue (*n* = 4 in each group) were homogenized by an electric homogenizer in radioimmunoprecipitation (RIPA) lysis buffer (P0013B, Beyotime) supplemented with phenylmethylsulphonyl fluoride (PMSF, ST506, Beyotime) and protease phosphatase inhibitor mixtures (P1045, Beyotime) for 2 h at 4 °C. The homogenate was centrifuged for 20 min (16,000 rpm, 4 °C). The supernatant was collected as a whole-cell protein extract and protein concentrations were determined using a bicinchoninic acid (BCA) protein assay kit (P0010, Beyotime). Equal amounts of protein were loaded onto a sodium dodecyl sulfate-polyacrylamide gel electrophoresis (SDS-PAGE) gel, electrophoresed, and transferred to polyvinylidene difluoride (PVDF) membranes. The membranes were then blocked with 5% nonfat milk for 2 h at 37 °C and incubated with rabbit anti-GFAP antibody (1:5000, ab207165, Abcam) and rabbit anti-glyceraldehyde-3-phosphate dehydrogenase (anti-GAPDH) antibody (1:2000, ab8245, Abcam) overnight at 4 °C. The membranes were washed with tris-buffered saline Tween-20 buffer (TBST) three times and incubated with horseradish peroxidase (HRP) labeled goat anti-rabbit IgG (H + L) (1:1000, A0208, Beyotime) for 1 h at 37 °C. The immunoblots were probed with western blot detection kits (BeyoECL Plus, P0018S, Beyotime, China) and visualized with an imaging system (Tanon-5200, China). GAPDH was used as a loading control.

### RNAscope in situ hybridization (ISH) assay

We examined the transcriptional levels of GFAP through an RNAscope 2.5 HD Duplex Reagent Kit (322430, Advanced Cell Diagnostics, ACD). 10-μm-thick frozen sections of the TNC tissue (*n* = 4 in each group) were fixed with 4% paraformaldehyde and dehydrated with 50%, 70%, and 100% concentrations of ethanol step by step. The detection of GFAP and Iba1 in the TNC tissue sections was performed following the manufacturer’s protocols using GFAP probe (313211, ACD) and Iba1 probe (319141-C2, ACD). We also used the positive control probe (PPIB-C1/POLR2A-C2, 321651, ACD) and 2-plex negative control probe (320751, ACD) as the positive and negative control, respectively. The images (× 40 objective) were captured under a light microscope (BX43, Olympus) using the cellSens standard software (version 1.18, Olympus). The numbers of blue (GFAP) and red (Iba1) dots and total cell numbers were calculated using ImageJ software. The mRNA level was presented as dots number per cell.

### Quantification detection of cytokines

We quantitatively detected 13 cytokines (including CCL2, CCL5, CCL7, CCL12, CXCL1, CXCL13, IFN-γ, TNF-α, M-CSF, IL-1β, IL-6, IL-10, IL-17A) in serum and TNC tissue extraction (*n* = 4 in each group) using Luminex Multiplex Immunoassays (R&D systems) according to the manufacture’s protocols. In brief, serum and the TNC tissue extraction were added to a 96-well microplate and mixed with a microparticle cocktail that was already coated with analyte-specific capture antibodies. After 2-h incubation at RT, the biotin-antibody cocktail was added to form an antibody-antigen sandwich in each bead. Subsequently, streptavidin-phycoerythrin was added to bind the biotinylated detection antibodies. Beads were read on a Luminex 100/200 system (Bio-Rad).

### Statistical analysis

Data were expressed as the mean ± standard error of mean (SEM). SPSS 25.0 was used for statistical analysis. Statistical differences between two groups were analyzed using the independent-sample t test. Multiple comparisons were statistically analyzed by one-way analysis of variance (ANOVA) and Dunnett’s t test post hoc analysis. *P* < 0.05 was considered statistically significant.

## Results

### Recurrent IS infusion induces cutaneous allodynia

**Fig. 2:**
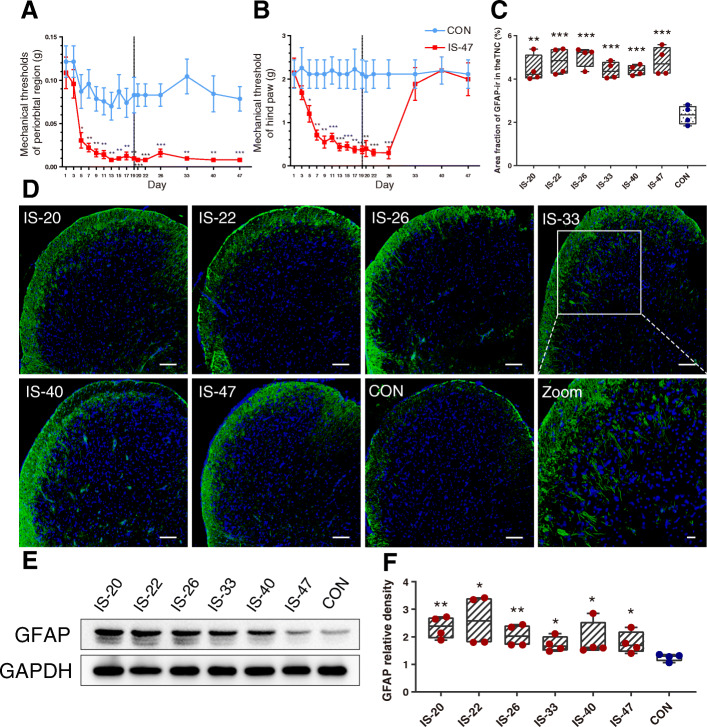
The effect of recurrent IS infusion on mechanical thresholds, immunofluorescent staining, and protein levels of GFAP in the TNC. **A, B** Mechanical thresholds in the periorbital and hind paw plantar regions of mice were assessed in the IS-47 group and CON group. Independent samples t test. *n* = 8/group. *p < 0.05, **p < 0.01, ***p < 0.001 versus CON. **C** The box plot presents the mean area fraction covered by GFAP-immunoreactive staining in the TNC of different groups (IS-20, IS-22, IS-26, IS-33, IS-40, IS-47, CON). One-way ANOVA and Dunnett’s t test post hoc analysis. *n* = 4/group, ** < 0.01, ***p < 0.001 versus CON. **D** Representative images of GFAP-immunoreactive staining in the TNC of different groups. The white box was enlarged to the zoom image (the last one). Scale bar = 100 μm. Green: GFAP. Blue: DAPI. **E** Representative images of Western blot results for GFAP in the TNC of different groups. **F** The box plot presents the quantitative analysis of GFAP. One-way ANOVA and Dunnett’s t test post hoc analysis. *n* = 4/group, *p < 0.05 versus CON, **p < 0.01 versus CON

To measure the variation with time of cutaneous allodynia of the animals, we use the vFF to examine the changes of mechanical thresholds in the periorbital region and hind paw plantar in the IS-47 and CON groups (*n* = 8/group) at a serial timeline (days 1, 3, 5, 7, 9, 11, 13, 15, 17, 19, 20, 22, 26, 33, 40, 47). In the period of constructing the animal model (10 times of IS/NS infusions, days 1–19), the mechanical thresholds in both the periorbital region and hind paw plantar in IS group had a downward trend compared with the CON group. Between 5 d and 19 d, the mechanical thresholds in the periorbital region and hind paw plantar in the IS group decreased significantly compared with the CON group (Fig. 2A, B). After all the 10 times IS/NS infusions were successfully implemented, the mechanical thresholds in the periorbital region in the IS group decreased significantly between 20 d (1 d after the last infusion) and 47 d (28 d after the last infusion) compared with the CON group (Fig. 2A). The mechanical thresholds in the hind paw plantar in the IS group decreased significantly between 20 d and 26 d (7 d after the last infusion) compared with the CON group (Fig. 2B).

### Recurrent IS infusion increases the protein levels of GFAP in the TNC

To investigate whether the astrocytes in the TNC are activated in IS groups and when the astrocytes became activated, we examined the immunofluorescent staining and western blot assay of GFAP in the TNC in different groups (groups IS-20, IS-22, IS-26, IS-33, IS-40, IS-47, CON, *n* = 4/group).

Immunofluorescence area fraction of GFAP-immunoreactive signals in all IS groups significantly increased compared with the CON group, indicating the GFAP-immunoreactive signals increased from 20 d and sustained at least 28 d to 47 d. And there were no significant differences among different IS groups (Fig. 2C, D).

The western blot results showed that the protein levels of GFAP in all IS groups significantly increased compared with the CON group, indicating the protein levels of GFAP increased from 20 d and sustained at least 28 d to 47 d. And there were no significant differences among different IS groups (Fig. 2E, F).

### Recurrent IS infusion increases the mRNA levels of GFAP and Iba1 in the TNC

**Fig. 3:**
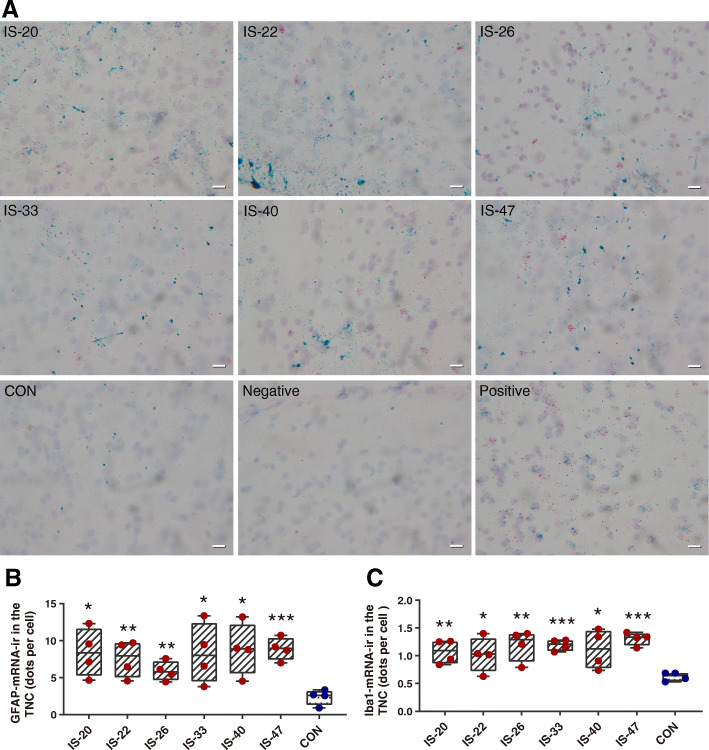
The mRNA levels of GFAP and Iba1 in the TNC. **A** Representative images of RNAscope ISH results for GFAP and Iba1 in the TNC of different groups. Blue dots: GFAP. Red dots: Iba1. Purple: cell nucleus. Scale bar: 20 μm. **B, C** The histograms present the quantitative analysis of the mRNA levels of GFAP and Iba1 in the TNC. One-way ANOVA and Dunnett’s t test post hoc analysis. *n* = 4/group, *p < 0.05, **p < 0.01, ***p < 0.001 versus CON

To verify the change of GFAP expression, we used the RNAscope assay to investigate the mRNA levels of GFAP in the TNC in different groups (groups IS-20, IS-22, IS-26, IS-33, IS-40, IS-47, CON, *n* = 4/group). We tested the mRNA levels of Iba1 as well. The results showed that the mRNA levels of GFAP and Iba1 in all IS groups significantly increased compared with the CON group, indicating the mRNA levels of GFAP and Iba1 increased from 20 d and sustained at least 28 d to 47 d. And there were no significant differences among different IS groups (Fig. 3A, B, C).

### Recurrent IS infusion changes the levels of CCL7, CCL12, and IL-10 in the TNC tissue extraction

**Fig. 4:**
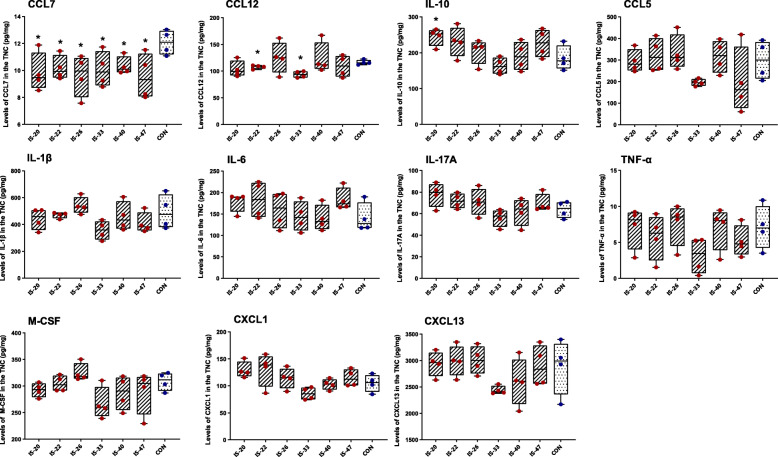
The levels of cytokines in the TNC tissue extraction of different groups. The box plots present the quantitative analysis of 11 cytokines (CCL7, CCL12, IL-10, CCL5, IL-1β, IL-6, IL-17A, TNF-α, M-CSF, CXCL1, and CXCL13). One-way ANOVA and Dunnett’s t test post hoc analysis. *n* = 4/group, *p < 0.05, **p < 0.01 versus CON

To further explore the correlation between glial activation and cytokine release in the TNC, we used Luminex immunoassays to investigate whether the levels of 13 cytokines in the TNC tissue extraction change. The results showed that the levels of CCL7 in all IS groups significantly decreased compared with the CON group, indicating the CCL7 levels decreased from 20 d and sustained at least 28 d to 47 d. We also found that the levels of CCL12 in the IS-22 (22 d) and IS-33 group (33 d) significantly decreased compared with the CON group. The levels of IL-10 in the IS-20 group (20 d) significantly increased compared with the CON group. The levels of CCL2 and IFN-γ were below the assay’s detection range. All the other examined cytokines were not significantly different between each IS group and the CON group (Fig. 4).

### Recurrent IS infusion changes the levels of CCL7, CCL12 in serum

**Fig. 5:**
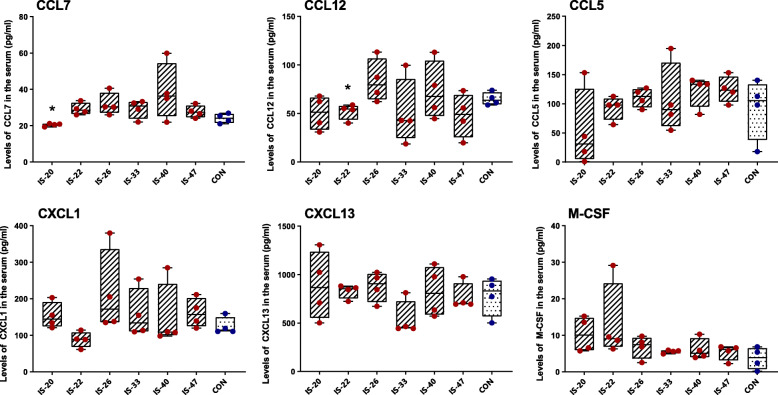
The levels of cytokines in serum of different groups. The box plots present the quantitative analysis of 6 cytokines (CCL7, CCL12, CCL5, CXCL1, CXCL13, and M-CSF). One-way ANOVA and Dunnett’s t test post hoc analysis. *n* = 4/group, *p < 0.05 versus CON

To further explore the correlation between glial activation and cytokine release in serum, we examined the same 13 cytokines in serum. The results showed that the levels of CCL7 in the IS-20 group (20 d) and the levels of CCL12 in the IS-22 group (22 d) significantly decreased compared with the CON group. The levels of CCL2, IFN-γ, IL-6, IL-17A, TNF-α, IL-1β, and IL-10 were below the assay’s detection range. The levels of all the other four examined cytokines (CCL5, CXCL1, CXCL13, M-CSF) in each IS group was not significantly different compared with the CON group (Fig. 5).

## Discussion

Accumulating evidence supports that central sensitization of the trigeminovascular system plays a significant role in CM. Astrocytes are critical in the central sensitization of chronic pain. Activation of astrocytes in the first order of trigeminovascular system (trigeminal ganglion, TG) in experimental CM, with the release of various cytokines (like IL-1β, IL-6, and TNF-α), causes cutaneous allodynia [3, 17]. But it is still unclear what the temporal characteristics of astrocytic activation are in the TNC, what cytokines are released in the TNC, and what the correlation is between the cytokines release and glial activation.

The recent study demonstrated that astrocytic activation in the TNC is related to the experimental CM [18]. After repeated dural IS infusion, astrocytes in the TNC proliferated and hypertrophied [18]. The cytokines released to the intercellular space may promote the crosstalk between the TNC neurons and neuroglia, and may lead to the central sensitization of the trigeminovascular system [19]. Pretreatment with ibudilast (a potential glial attenuator) prevented the development of facial allodynia in a dural IS-induced rats model [20]. However, there remained debate about whether glial cells are potential targets of clinical treatments. A recent clinical pilot trial about the efficacy of oral usage of ibudilast in 14 CM patients showed no efficacy [21]. Our data provide further evidence that astrocytes in the TNC are activated and may play a crucial role in the central sensitization in a mice model of pain induced by recurrent dural IS infusion.

In this study, we successfully establish the mice model of experimental migraine induced by dural IS infusion. The mice model showed time-dependent and reversible cutaneous allodynia. The cutaneous allodynia was sustained several weeks after the last infusion, which was consistent with the clinical features of migraine [5, 7, 22]. The allodynia in the periorbital region of mice sustained at least 28 d, which may indicate the sensitization of the trigeminovascular system, including TG, TNC, and even ventral posteromedial nucleus (VPM) in the thalamus. The allodynia in the hind paw plantar sustained at least 14 d. The algesia in the hind paw plantar conducts through the spinal ganglion, spinal dorsal horn neurons, and ventral posterolateral nucleus (VPL) in the thalamus. But in this animal model, there was no direct noxious stimulus to the signaling pathway of algesia in the hind paw plantar, and the vFF test showed no result in hyperalgesia in that region (CON group). Therefore, the allodynia in the hind paw plantar may suggest the sensitization of VPL in the thalamus, which is possibly induced by the sensitization of VPM through some mechanisms, such as glia-neuron and glia-glia communication. The difference of the allodynia duration in the periorbital region and hind paw plantar represents a varied duration of sensitization among TG, TNC, VPM, and VPL.

Consistent with the previous study, recurrent IS infusion induced the activation of astrocytes in the TNC [23]. But the previous study did not demonstrate the temporal characteristics of astrocytic activation in the TNC. In this study, GFAP, a biomarker of astrocytic activation, was detected from 20 d to 47 d (1d to 28 d after the last infusion) to examine the temporal characteristics of astrocytic activation in the TNC. We detected the GFAP in the TNC at the protein level and transcription level at different time points (days 20, 22, 26, 33, 40, 47), including immunofluorescence staining, western blot assay, and RNAscope ISH assay. The results suggested that the astrocytes in the TNC were activated from 20 d to at least 47 d. We also detected Iba1 in the TNC, a biomarker of microglial activation, at transcription level through RNAscope ISH, and the results suggested that the microglia were activated at the same time with astrocytes. Microglia may contribute to the central sensitization through modulating cytokine signals communication among the astrocytes, microglia, and neurons [12, 24–27]. The duration of astrocytic and microglial activation is consistent with the sensitization of the trigeminovascular system. It is noted that only male mice were used in the research and the consistency of the results on female mice needs to be further explored.

Neurogenic neuroinflammation caused by the repeated stimulus of neurons and continuous release of cytokines could be central to the understanding of migraine chronification [3]. Animal experiments support this idea, but clinical studies have yet to be conducted [28]. The idea of neurogenic neuroinflammation in the trigeminal ganglion could explain the findings of inflammatory markers in migraine [29, 30]. Enhance of cytokines production and release in the trigeminal ganglion could lead to sensitization [31]. But few studies are aiming at the levels change of cytokines in the TNC.

With the astrocytic activation in the TNC, cytokines are released into the extracellular environment near the neurons and astrocytes. In clinical studies, it has been found that the levels in serum of IL-6 and TNF-α in migraineurs are higher than those in normal people, suggesting that neuroinflammation plays a certain role in the pathological mechanism of migraine [32]. But this finding has not been verified in animal experiments. In this study, we quantitatively detected 13 cytokines, which may be associated with migraine, in TNC tissue extraction and serum of mice. In the TNC tissue extraction, the results showed that the CCL7 levels in IS groups were decreased from 20 d to 47 d. We also found that the CCL12 levels decreased on 22 d and 33 d, and the IL-10 levels increased on 20 d. However, there were no differences in the levels of IL-1 β, IL-6, IL-17A, TNF-α, M-CSF, CCL5, CXCL1, CXCL13. Notably, the levels of CCL2 and IFN-γ are below the detection range, which means an uncertain correlation of the two cytokines between each IS group and CON group. In serum, the results showed that the CCL7 levels in IS groups decreased on 20 d, and the CCL12 levels decreased on 22 d. However, we did not find differences in CCL5, CXCL1, CXCL13, and M-CSF between each IS group and the CON group, and the levels of CCL2, IFN-γ, IL-6, IL-17A, TNF-α, IL-1β, and IL-10 are below the detection range. We think the differences between animal experiments and clinical studies in cytokines release are caused by the limitation of current animal models. The present animal models could only partially simulate the pathological process and clinical features of CM. Though having a larger dataset would offer further insight, this study could demonstrate that the decreased expression of CCL7 and CCL12, and increased expression of IL-10 may correlate with the pathophysiology of repeated dural IS-induced pain through astrocytic and microglial activation in the TNC.

CCL7 or monocyte chemotactic protein 3 (MCP-3), a secreted chemokine that attracts macrophages during inflammation and metastasis. It is a member of the C-C subfamily of chemokines which are characterized by having two adjacent cysteine residues. The protein is an in vivo substrate of matrix metalloproteinase 2, an enzyme that degrades components of the extracellular matrix. CCL12 or monocyte chemotactic protein 5 (MCP-5), is another member of the C-C subfamily of chemokines [33, 34]. The chemokines CCL7 and CCL12 are known to promote the recruitment of many innate immune cell types including monocytes and neutrophils to sites of bacterial and viral infection and eosinophils and basophils to sites of allergic inflammation [35]. The difference of duration of the downregulation of CCL7 and CCL12 levels in the TNC tissue extraction and serum may indicate that CCL7 and CCL12 may release by astrocytes or other gliocytes in the TNC, not by cells in the blood or other tissue. IL-10 is an anti-inflammatory cytokine, which targets immune responses and exerts immunosuppressive functions [36]. In the TNC, some phenotypes of microglia could be suppressed by anti-inflammatory cytokines such as IL-10, IL-4, and substance P [26]. Regardless of other incident factors, the downregulation of CCL7 and CCL12, together with the upregulation of IL-10, may demonstrate the dynamic balance of neuroinflammation and anti-inflammation, and may suggest the correlation between neuroinflammation and IS-induced pain in a period of time.

## Conclusion

Our findings indicate that the astrocytic activation in the TNC correlates to the central sensitization in the mice model of pain induced by recurrent dural IS infusion. The temporal characteristics of astrocytic activation in the TNC are that the astrocytes are activated from 20 d to 47 d and there are no significant differences in the activation levels. CCL7, CCL12, and IL-10 may correlate with astrocytic or microglial activation in the mice model. Some cytokines (such as IL-1β, IL-6, and TNF-α), which were previously suggested the correlation to CM, do not show significant findings in this study.

## Data Availability

The datasets generated and analyzed during the current study are available from the corresponding author on reasonable request.

## Abbreviations

CM: Chronic migraine
TNC: Trigeminal nucleus caudalis
GFAP: Glial fibrillary acidic protein
IL-1β: Interleukin 1 beta
IL-6: Interleukin 6
TNF-α: Tumor necrosis factor alpha
IS: Inflammatory soup
mRNA: Messenger ribonucleic acid
Iba1: Ionized calcium binding adapter molecule 1
CCL2: C-c motif chemokine ligand 2
CCL5: C-c motif chemokine ligand 5
CCL7: C-c motif chemokine ligand 7
CCL12: C-c motif chemokine ligand 12
CXCL1: C-x-c motif chemokine ligand 1
CXCL13: C-x-c motif chemokine ligand 13
IFN-γ: Interferon gamma
M-CSF: Macrophage colony-stimulating factor
IL-10: Interleukin 10
IL-17A: Interleukin 17A
NS: Normal saline
CON: Control
vFF: von Frey filaments
EP: Eppendorf
PBS: Phosphate buffered solution
OCT: Optimum cutting temperature compound
RT: Room temperature
DAPI: 4′,6-diamidino-2-phenylindole
RIPA: Radioimmunoprecipitation
PMSF: Phenylmethylsulphonyl fluoride
BCA: Bicinchoninic acid
SDS-PAGE: Sodium dodecyl sulphate-polyacrylamide gel electrophoresis
PVDF: Polyvinylidene difluoride
GAPDH: Glyceraldehyde-3-phosphate dehydrogenase
TBST: Tris-buffered saline Tween-20 buffer
HRP: horseradish peroxidase
ISH: In situ hybridization
SEM: Standard error of mean
ANOVA: Analysis of variance
TG: Trigeminal ganglion
VPM: Ventral posteromedial nucleus
VPL: Ventral posterolateral nucleus
MCP-3: Monocyte chemotactic protein 3
MCP-5: Monocyte chemotactic protein 5

## References

[1] Vos T, Abajobir AA, Abbafati C (2017). Global, regional, and national incidence, prevalence, and years lived with disability for 328 diseases and injuries for 195 countries, 1990-2016: a systematic analysis for the global burden of disease study 2016. Lancet.

[2] Su M, Yu S (2018). Chronic migraine: a process of dysmodulation and sensitization. Mol Pain.

[3] Edvinsson L, Haanes KA, Warfvinge K (2019). Does inflammation have a role in migraine?. Nat Rev Neurol.

[4] Andreou AP, Edvinsson L (2019). Mechanisms of migraine as a chronic evolutive condition. J Headache Pain.

[5] Edelmayer RM, Vanderah TW, Majuta L, Zhang ET, Fioravanti B, de Felice M, Chichorro JG, Ossipov MH, King T, Lai J, Kori SH, Nelsen AC, Cannon KE, Heinricher MM, Porreca F (2009). Medullary pain facilitating neurons mediate allodynia in headache-related pain. Ann Neurol.

[6] Goadsby PJ, Holland PR, Martins-Oliveira M, Hoffmann J, Schankin C, Akerman S (2017). Pathophysiology of migraine: a disorder of sensory processing. Physiol Rev.

[7] Louter MA, Bosker JE, Van Oosterhout WPJ (2013). Cutaneous allodynia as a predictor of migraine chronification. Brain.

[8] Lukács M, Warfvinge K, Tajti J, Fülöp F, Toldi J, Vécsei L, Edvinsson L (2017) Topical dura mater application of CFA induces enhanced expression of c-fos and glutamate in rat trigeminal nucleus caudalis: attenuated by KYNA derivate (SZR72). J headache pain 18. 18(1):39. 10.1186/s10194-017-0746-x 10.1186/s10194-017-0746-x PMC5364126 28337634

[9] Romero-Reyes M, Pardi V, Akerman S (2015). A potent and selective calcitonin gene-related peptide (CGRP) receptor antagonist, MK-8825, inhibits responses to nociceptive trigeminal activation: role of CGRP in orofacial pain. Exp Neurol.

[10] Perini F, D’Andrea G, Galloni E (2005). Plasma cytokine levels in migraineurs and controls. Headache.

[11] Kang L, Tang W, Zhang Y, Zhang M, Liu J, Li Y, Kong S, Zhao D, Yu S (2021) The gut microbiome modulates nitroglycerin-induced migraine-related hyperalgesia in mice. Cephalalgia.:033310242110500. 10.1177/03331024211050036 10.1177/03331024211050036 34644194

[12] Jing F, Zou Q, Wang Y, Cai Z, Tang Y (2021). Activation of microglial GLP-1R in the trigeminal nucleus caudalis suppresses central sensitization of chronic migraine after recurrent nitroglycerin stimulation. J Headache Pain.

[13] Oshinsky ML, Gomonchareonsiri S (2007). Episodic dural stimulation in awake rats: a model for recurrent headache. Headache.

[14] Su M, Ran Y, Han X, Liu Y, Zhang X, Tan Q, Li R, Yu S (2016). Rizatriptan overuse promotes hyperalgesia induced by dural inflammatory stimulation in rats by modulation of the serotonin system. Eur J Neurosci.

[15] Trevisan dos Santos G, Jacobs B, Asiedu M (2018). Non-invasive dural stimulation in mice: A novel preclinical model of migraine. Cephalalgia.

[16] Fried NT, Maxwell CR, Elliott MB, Oshinsky ML (2018). Region-specific disruption of the blood-brain barrier following repeated inflammatory dural stimulation in a rat model of chronic trigeminal allodynia. Cephalalgia.

[17] Su M, Ran Y, He Z, Zhang M, Hu G, Tang W, Zhao D, Yu S (2018). Inhibition of toll-like receptor 4 alleviates hyperalgesia induced by acute dural inflammation in experimental migraine. Mol Pain.

[18] Zhou X, Liang J, Wang J, Fei Z, Qin G, Zhang D, Zhou J, Chen L (2019). Up-regulation of astrocyte excitatory amino acid transporter 2 alleviates central sensitization in a rat model of chronic migraine. J Neurochem.

[19] He W, Long T, Pan Q, Zhang S, Zhang Y, Zhang D, Qin G, Chen L, Zhou J (2019). Microglial NLRP3 inflammasome activation mediates IL-1β release and contributes to central sensitization in a recurrent nitroglycerin-induced migraine model. J Neuroinflammation.

[20] Wieseler J, Ellis A, McFadden A, Stone K, Brown K, Cady S, Bastos LF, Sprunger D, Rezvani N, Johnson K, Rice KC, Maier SF, Watkins LR (2017) Supradural inflammatory soup in awake and freely moving rats induces facial allodynia that is blocked by putative immune modulators. Brain Res:1664–1694. 10.1016/j.brainres.2017.03.011 10.1016/j.brainres.2017.03.011 PMC6668623 28322750

[21] Kwok YH, Swift JE, Gazerani P, Rolan P (2016). A double-blind, randomized, placebo-controlled pilot trial to determine the efficacy and safety of ibudilast, a potential glial attenuator, in chronic migraine. J Pain Res.

[22] Landy SH, McGinnis JE, McDonald SA (2007). Clarification of developing and established clinical allodynia and pain-free outcomes. Headache.

[23] Raghavendra V, Tanga FY, DeLeo JA (2004). Complete Freunds adjuvant-induced peripheral inflammation evokes glial activation and proinflammatory cytokine expression in the CNS. Eur J Neurosci.

[24] Linnerbauer M, Wheeler MA, Quintana FJ (2020). Astrocyte crosstalk in CNS inflammation. Neuron.

[25] Gong Q, Lin Y, Lu Z, Xiao Z (2020). Microglia-astrocyte cross talk through IL-18/IL-18R signaling modulates migraine-like behavior in experimental models of migraine. Neuroscience..

[26] Kim S, Son Y (2021) Astrocytes stimulate microglial proliferation and m2 polarization in vitro through crosstalk between astrocytes and microglia. Int J Mol Sci 22(16). 10.3390/ijms22168800 10.3390/ijms22168800 PMC8396240 34445510

[27] Long T, He W, Pan Q, Zhang S, Zhang D, Qin G, Chen L, Zhou J (2020). Microglia P2X4R-BDNF signalling contributes to central sensitization in a recurrent nitroglycerin-induced chronic migraine model. J Headache Pain.

[28] Zhang L, Kunkler PE, Knopp KL, Oxford GS, Hurley JH (2019). Role of intraganglionic transmission in the trigeminovascular pathway. Mol Pain.

[29] Haanes KA, Edvinsson L (2019). Pathophysiological mechanisms in migraine and the identification of new therapeutic targets. CNS Drugs.

[30] Melo-Carrillo A, Strassman AM, Nir RR, Schain AJ, Noseda R, Stratton J, Burstein R (2017). Fremanezumab—a humanized monoclonal anti-cgrp antibody—inhibits thinly myelinated (Aδ) but not unmyelinated (c) meningeal nociceptors. J Neurosci.

[31] Afroz S, Arakaki R, Iwasa T, Oshima M, Hosoki M, Inoue M, Baba O, Okayama Y, Matsuka Y (2019) CGRP induces differential regulation of cytokines from satellite glial cells in trigeminal ganglia and orofacial nociception. Int J Mol Sci 20(3). 10.3390/ijms20030711 10.3390/ijms20030711 PMC6386987 30736422

[32] Martami F, Razeghi Jahromi S, Togha M, Ghorbani Z, Seifishahpar M, Saidpour A (2018). The serum level of inflammatory markers in chronic and episodic migraine: a case-control study. Neurol Sci.

[33] Popiolek-Barczyk K, Ciechanowska A, Ciapała K, Pawlik K, Oggioni M, Mercurio D, de Simoni MG, Mika J (2020). The CCL2/CCL7/CCL12/CCR2 pathway is substantially and persistently upregulated in mice after traumatic brain injury, and CCL2 modulates the complement system in microglia. Mol Cell Probes.

[34] Jaerve A, Müller HW (2012). Chemokines in CNS injury and repair. Cell Tissue Res.

[35] Xue J, Zhang Y, Zhang J, Zhu Z, Lv Q, Su J (2021). Astrocyte-derived CCL7 promotes microglia-mediated inflammation following traumatic brain injury. Int Immunopharmacol.

[36] Ouyang W, O’Garra A (2019). IL-10 family cytokines IL-10 and IL-22: from basic science to clinical translation. Immunity.

